# Comparison of renal region, cerebral and peripheral oxygenation for predicting postoperative renal impairment after CABG

**DOI:** 10.1007/s10877-021-00701-4

**Published:** 2021-04-20

**Authors:** Ilonka N. de Keijzer, Marieke Poterman, Anthony R. Absalom, Jaap Jan Vos, Massimo A. Mariani, Thomas W. L. Scheeren

**Affiliations:** 1 Department of Anaesthesiology, University Medical Centre Groningen, Hanzeplein 1, Groningen, 9713 GZ The Netherlands; 2 Department of Cardiothoracic Surgery, University Medical Centre Groningen, Groningen, The Netherlands

**Keywords:** Coronary artery bypass grafting (CABG), Renal oxygenation, Cerebral oxygenation, Acute kidney injury, Postoperative renal impairment

## Abstract

Patients undergoing coronary artery bypass grafting (CABG) are at risk of developing postoperative renal impairment, amongst others caused by renal ischemia and hypoxia. Intra-operative monitoring of renal region tissue oxygenation (SrtO_2_) might be a useful tool to detect renal hypoxia and predict postoperative renal impairment. Therefore, the aim of this study was to assess the ability of intra-operative SrtO_2_ to predict postoperative renal impairment, defined as an increase of serum creatinine concentrations of  > 10% from individual baseline, and compare this with the predictive abilities of peripheral and cerebral tissue oxygenation (SptO_2_ and SctO_2_, respectively) and renal specific tissue deoxygenation. Forty-one patients undergoing elective CABG were included. Near-infrared spectroscopy (NIRS) was used to measure renal region, peripheral (thenar muscle) and cerebral tissue oxygenation during surgery. Renal region specific tissue deoxygenation was defined as a proportionally larger decrease in SrtO_2_ than SptO_2_. ROC analyses were used to compare predictive abilities. We did not observe an association between tissue oxygenation measured in the renal region and cerebral oxygenation and postoperative renal impairment in this small retrospective study. In contrast, SptO_2_ decrease > 10% from baseline was a reasonable predictor with an AUROC of 0.767 (95%CI  0.619 to 0.14; p = 0.010). Tissue oxygenation of the renal region, although non-invasively and continuously available, cannot be used in adults to predict postoperative renal impairment after CABG. Instead, peripheral tissue deoxygenation was able to predict postoperative renal impairment, suggesting that SptO_2_ provides a better indication of ‘general’ tissue oxygenation status.

Registered at ClinicalTrials.gov: NCT01347827, first submitted April 27, 2011.

## Introduction

Cardiac surgery is often complicated by acute kidney injury (AKI). The incidence of AKI after cardiac surgery, as identified by commonly used AKI classification systems (AKIN, KDIGO, or RIFLE) varies from 5 to 42% depending on comorbidity, surgical technique, and the population studied [1, 2]. Even minor renal impairment—not identified by these AKI classifications—is associated with increased postoperative morbidity and mortality after cardiac surgery [3, 4]. In the time since the AKI classifications have become widely accepted, studies have been published that suggest that smaller changes in serum creatinine should not be ignored [5, 6]. Therefore it has been advocated that impaired postoperative renal function is best evaluated as an increase of postoperative serum creatinine levels compared to individual preoperative levels [4, 7]**.**

It remains difficult to properly predict who is at risk for developing postoperative renal impairment after cardiac surgery, especially when cardiopulmonary bypass (CPB) is used, as it can cause haemodynamic alterations and possibly disturb renal autoregulation [8]. Intra-operative tissue oxygenation measured in the renal region (SrtO_2_) offers a non-invasive dynamic insight into tissue oxygenation, by assessing the balance between oxygen delivery and oxygen consumption at the tissue level [9], and may serve to identify patients at risk of renal function loss. SrtO_2_ was correlated to renal venous oxygen saturation (r_rm_ = 0.61, p < 0.001) in adult on-pump cardiac surgery patients [10] and in paediatric patients renal tissue deoxygenation has been shown to be associated with adverse postoperative outcomes [11–15]. However, whether SrtO_2_ also predicts postoperative impairment of renal function in adult cardiac surgery patients, is unknown.

In the current study we hypothesized that tissue deoxygenation of the renal region during coronary artery bypass grafting (CABG) is a good predictor of postoperative renal impairment in adult patients with normal preoperative serum creatinine concentrations. Additionally, the ability of tissue deoxygenation of the renal region to predict postoperative renal impairment was compared with that of peripheral and cerebral tissue oxygenation (SptO_2_ and SctO_2_, respectively) and renal region specific tissue deoxygenation (defined as a proportionally larger decrease in SrtO_2_ than SptO_2_).

## Material and methods

This study is a secondary analysis of data from a randomized controlled trial comparing the incidence and severity of *cerebral* oxygen desaturation during CABG, with and without CPB [16]. Data were obtained in 59 patients who were randomized to on-pump or off-pump CABG and who had near-infrared spectroscopy (NIRS) sensors applied at the flank, the thenar muscle, and the forehead to measure renal region, peripheral, and cerebral tissue oxygenation, respectively. The study was performed at the University Medical Centre Groningen, The Netherlands, between June 2011 and May 2012. Ethical approval (Reference: METc2011/045) was obtained from the institutional ethical committee, Groningen, The Netherlands. The study was registered at ClinicalTrials.gov (NCT01347827).

### Study procedures and patient selection

The details of the study protocol and intra-operative anaesthetic management have been published previously [16]. In brief, patients aged 18 years and older were included when both the operating surgeon and the responsible anaesthesiologist considered the patient eligible for CABG performed either with or without CPB. Exclusion criteria included pre-existing acute or chronic renal impairment (serum creatinine concentration above 200 µmol^.^l^−1^).

### Determination of renal impairment

We defined renal impairment as an increase of serum creatinine concentrations of > 10% from individual baseline values within the first 7 days postoperatively.

### Oxygenation measurement

During CABG, tissue oxygenation was measured non-invasively and continuously by NIRS with the INVOS 5100 Oximeter (Covidien, Dublin, Ireland (now part of Medtronic, Minneapolis, Minnesota (USA)). After induction of anaesthesia, an ultrasound examination of the left and right superior lumbar region was performed by the anaesthesiologist to determine the location of the kidneys and their depth beneath the skin surface. Assessment of renal tissue oxygenation was only considered feasible in patients where the kidney surface was located less than 5 cm from skin level. Sensors were applied to the skin of the superior lumbar regions to assess SrtO_2_ of both kidneys separately. Cerebral oxygenation was monitored using NIRS monitors and sensors from two manufacturers, but for this study we only used the oxygenation measurements from the INVOS Cerebral Oximeter 5100C (Covidien, Dublin, Ireland (now part of Medtronic, Minneapolis, Minnesota (USA)). Another sensor was placed on the right thenar eminence to measure peripheral tissue oxygenation with an InSpectra™ device (Hutchinson Technology, Hutchinson, USA).

### Calculation of oxygenation data

Baseline renal region, cerebral and peripheral tissue oxygenation data were obtained after induction of anaesthesia with the lungs of the patient being ventilated with an inspiratory oxygen fraction (FiO_2_) of 0.4 and were calculated as the mean value of SrtO_2_, SctO_2_ and SptO_2_ during the 5 min before surgical incision. The area under the threshold (AUT) of renal region and cerebral tissue deoxygenation was quantified for absolute and relative thresholds of SrtO_2_ and SctO_2_ in %min. For absolute values we applied thresholds of 90%, 80%, 70%, and 60%. For relative changes, we calculated AUTs for thresholds that were 5%, 10%, and 15% below each individual’s baseline. For SptO_2_ only the AUT for a relative threshold of > 10% decrease from baseline SptO_2_ was assessed.

Oxygenation data below each threshold were expressed as the AUT (in %min) below each threshold, and were calculated using the following formulae:

- for absolute thresholds: AUT < 90% = (90−current SrtO_2_) × time (minutes)
- for relative thresholds: AUT > 5% below baseline SrtO_2_ = (0.95 × baseline SrtO_2_–current SrtO_2_) × time (minutes).

**Fig. 1:**
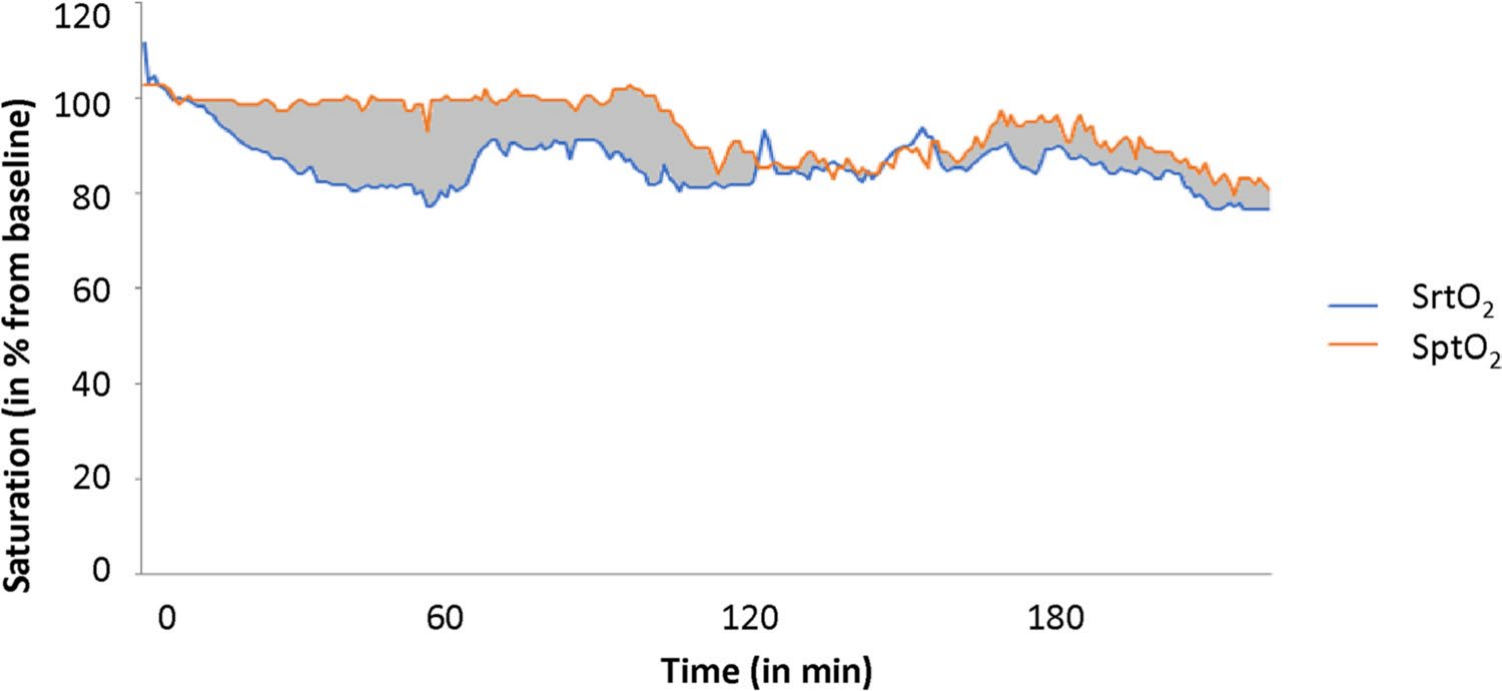
Example of the calculation of renal region specific tissue deoxygenation. The grey area is the area under the threshold (AUT; (%min)) of renal region specific tissue deoxygenation, which was calculated by subtracting the SrtO_2_ in % from baseline (renal tissue (blue line)) from the SptO_2_ in % from baseline (peripheral tissue (orange line))

The AUT of renal region specific tissue deoxygenation was calculated as follows: individual baseline SrtO_2_ and SptO_2_ were set at 100%. All intraoperative values were calculated as a percentage change from baseline. Subsequently, SrtO_2_ values (in % from baseline SrtO_2_) were subtracted from SptO_2_ (in % from baseline SptO_2_) and all positive values (when SptO_2_ > SrtO_2_) were summed up to assess the AUT of renal region specific tissue deoxygenation (Fig. 1).

### Statistical analysis

Statistical analyses were performed in SPSS version 23 (IBM Inc., Chicago, USA). Normality of continuous variables was assessed by the Shapiro–Wilk test. Continuous data are expressed as mean (SD) for parametric data or as median [IQR range] for non-parametric data. To compare continuous variables for parametric and non-parametric data, an independent *T* test or Mann–Whitney *U* test was performed respectively. Categorical variables are presented as number (proportion) and compared by using Fisher’s Exact or Chi-square test, depending on the number of patients per group. Correlation between left and right renal region tissue oxygenation measurements was assessed using Spearman’s correlation coefficient. Receiver operating characteristics (ROC) curves were created to assess the predictive value of absolute and relative oxygenation thresholds. Statistical significance was defined as p < 0.05.

## Results

### Patient characteristics

In the original study 59 patients were included of whom 18 did not have sufficient data for this secondary analysis. Reasons for exclusion were: no renal region tissue oxygenation data (n = 12), no peripheral tissue oxygenation data (n = 1), or insufficient renal region and peripheral tissue oxygenation data (n = 5). Renal region tissue oxygenation data were missing due to depth or the location of the kidneys, e.g. > 5 cm deep, or the kidney(s) could not be visualized on ultrasound imaging, and for peripheral tissue oxygenation NIRS sensors were not applied. Thus, 41 patients with a mean age of 63 (9) years remained for analysis: 19 patients who had been randomized to undergo CABG with CPB and 22 patients randomized to undergo CABG without CPB for the purpose of the original study [16]. All patients were classified as ASA physical status III. In 35 out of these 41 patients SrtO_2_ data were obtained bilaterally, and in these cases left and right SrtO_2_ values were averaged (mean correlation between left and right kidney of r = 0.80, p < 0.001). In 3 out of 41 cases cerebral oxygenation data was obtained bilaterally and the left and right SctO2 values were averaged. In 11 patients (27%) serum creatinine concentrations increased > 10% from baseline within the first 7 days postoperatively. Patients with postoperative renal impairment had a longer duration of surgery (197 [182–240] min vs. 173 (26) min, p = 0.008) and needed more grafts (4 [3, 4] vs. 3 [3], p = 0.009). Data describing demographic, intra-operative and postoperative patient characteristics are shown in Table 1.

**Table 1:** Demographic, intra-operative and postoperative characteristics in patients undergoing on-pump or off-pump CABG, compared by postoperative renal function. Variables are number (%), mean (SD) or median [IQR]

	All patients (*n* = 41)	Patients without renal impairment (n = 30)	Patients with renal impairment (n = 11)	P value
Gender; male	38 (93%)	27 (90%)	11 (100%)	0.551
Age; years	63 (9)	62 (9)	66 (9)	0.155
BMI; kg m^−2^	27.6 (3.4)	27.3 (3.2)	28.4 (4.0)	0.361
Operated on CPB (on-pump)	19 (46%)	12 (40%)	7 (64%)	0.290
EuroSCORE	1.8 [1.3–3.0]^1^	1.5 [1.0–2.9]	2.6 [1.6–3.3]	0.095
DM type II	10 (24%)	8 (27%)	2 (18%)	0.700
Hypertension	11 (27%)	7 (23%)	7 (64%)	0.445
Smoking	26 (63%)^2^	19 (63%)	7 (64%)	1.000
Pre-operative LVEF				0.349
55–70%	19 (46%)	16 (53%)	3 (27%)	
40–55%	19 (46%)	12 (40%)	7 (64%)	
< 40%	3 (7%)	2 (7%)	1 (9%)	
Preoperative Creatinine; µmol l^−1^	84 [75–103]	84 [76–94]	93 (28)	0.942
Baseline Hb; mmol l^−1^	8.3 (0.7)	8.4 [8.2–8.6]	8.2 (0.9)	0.851
Duration of surgery; min	180 [159–196]	173 (26)	197 [182–240]	0.008*
CPB time^a^; min	83 (22)	82 (20)	86 (26)	0.697
Aorta cross clamp time^a^; min	53 (14)	53 (13)	53 (15)	0.997
No. of grafts per patient	3 [3, 4]	3 [3–3]	4 [3, 4]	0.009*
Intra-operative analyses				
Lactate; highest	2.4 (0.9)	2.4 (0.8)	2.5 (1.1)	0.883
Mean intraoperative MAP; mmHg	70 (6)	70 (6)	71 (7)	0.574
Stay on ICU; days	1 [1–1]	1 [1–1]	1 [1–4]	0.315
Stay in hospital; days	8 [6–11]	8 (3)	11 (5)	0.052

^1^EuroSCORE is missing from 2 patients. ^2^Smoking is missing from 1 patient

^a^Only in on-pump patients. *BMI* body mass index, *CPB* cardiopulmonary bypass, *ASA* american society of anesthesiologist status, *EuroSCORE* European system for cardiac operative risk evaluation, *DM type II* diabetes mellitus type 2, *LVEF* left ventricular ejection fraction, *Hb* haemoglobin, *MAP* mean arterial pressure, *ICU* intensive care unit. Significance is indicated with * for p < 0.05

### Tissue oxygenation

Baseline SrtO_2_, SctO_2_ and SptO_2_ values of patients with and without postoperative renal impairment were similar (Table 2). The AUT below all predefined absolute and relative SrtO_2_ and SctO_2_ thresholds, and renal specific tissue deoxygenation did not show any significant differences between patients with and without postoperative renal impairment. However, the AUT SptO_2_ > 10% decrease from baseline was significantly higher in patients with postoperative renal impairment (215 [72–934] %min vs. 36 [0–313] %min, p = 0.009). In 8 patients no deoxygenation of peripheral tissue occurred, i.e. SptO_2_ did not drop below 90% from baseline, and none of those patients developed postoperative renal impairment. In a subgroup analysis, tissue oxygenation was compared between on- and off-pump CABG. No differences were found between subgroups in baseline SrtO_2_, SctO_2_ and SptO_2_ and all absolute and relative thresholds (Table 3). However, the extent of renal region specific tissue deoxygenation was higher in on-pump CABG compared to off-pump CABG (1015 [443–1784] %min vs. 327 [81–1044] %min, p = 0.025).

**Table 2:** Intra-operative oxygenation data of patients with and without postoperative renal impairment. Variables are mean (SD) or median [IQR]

	No renal impairment (n = 30)	Renal impairment (n = 11)	P value
SrtO_2_			
Baseline; %	80 [75–86]	77 (11)	0.571
AUT absolute decrease SrtO_2_; %min			
Below 90% SrtO_2_	2396 [1372–3347]	4333 (3539)	0.359
Below 80% SrtO_2_	779 [204–1408]	876 [115–5348]	0.407
Below 70% SrtO_2_	114 [12–275]	172 [54–1880]	0.287
Below 60% SrtO_2_	13 [0–53]	34 [0–285]	0.226
AUT relative decrease SrtO_2_; %min			
> 5% from baseline	319 [131–701]	297 [207–895]	0.717
> 10% from baseline	119 [29–358]	119 [63–502]	0.805
> 15% from baseline	46 [10–157]	54 [20–312]	0.761
AUT of renal region specific tissue deoxygenation; %min	720 [143–1293]	443 [116–1507]	0.532
SctO_2_			
Baseline; %	65 (10)	63 (10)	0.547
AUT absolute decrease SctO_2_; %min			
Below 90% SctO_2_	5984 (2219)	7697 (2020)	0.051
Below 80% SctO_2_	3710 (2066)	4989 (2491)	0.105
Below 70% SctO_2_	1092 [445–3253]	2520 (1938)	0.391
Below 60% SctO_2_	146 [16–996]	593 [109–1609]	0.315
AUT relative decrease SctO_2_; %min			
> 5% from baseline	177 [60–576]	289 [158–711]	0.571
> 10% from baseline	57 [13–255]	147 [41–287]	0.391
> 15% from baseline	21 [3–90]	67 [9–92]	0.375
SptO_2_			
Baseline; %	84 (7)	83 (6)	0.710
AUT 10% below baseline; %min	36 [0–313]	215 [72–934]	0.009*

*SrtO*_*2*_ renal region tissue oxygenation, *SctO*_*2*_ cerebral tissue oxygenation, *SptO*_*2*_ peripheral tissue oxygenation, *AUT* area under the threshold. Significance is indicated with * for p < 0.05

**Table 3:** Intra-operative oxygenation data of patients who underwent on-pump or off-pump CABG. Variables are mean (SD) or median [IQR]

	Off-pump (n = 22)	On-pump (n = 19)	P value
SrtO_2_			
Baseline; %	78 (10)	81 (9)	0.312
AUT absolute decrease SrtO_2_; %min			
Below 90% SrtO_2_	2218 [1302 – 3959]	2591 [1922–3583]	0.480
Below 80% SrtO_2_	752 [125–1700]	802 [389–1591]	0.433
Below 70% SrtO_2_	119 [8–348]	121 [51–301]	0.695
Below 60% SrtO_2_	19 [0–100]	10 [0–41]	0.463
AUT relative decrease SrtO_2_; %min			
> 5% from baseline	276 [74–500]	452 [238–911]	0.089
> 10% from baseline	107 [14–213]	166 [67–502]	0.182
> 15% from baseline	28 [10–123]	77 [13–267]	0.306
AUT of renal region specific tissue deoxygenation; %min	327 [81–1044]	1015 [443–1784]	0.025*
SctO_2_			
Baseline; %	65 (8)	63 (12)	0.612
AUT absolute decrease SctO_2_; %min			
Below 90% SctO_2_	6273 (2524)	6641 (2545)	0.645
Below 80% SctO_2_	3940 (2154)	4184 (2367)	0.733
Below 70% SctO_2_	1227 [666–3261]	1777 [420–3891]	0.948
Below 60% SctO_2_	159 [52–996]	193 [12–1609]	0.923
AUT relative decrease SctO_2_; %min			
> 5% from baseline	191 [86–537]	230 [44–652]	0.907
> 10% from baseline	57 [17- 268]	127 [14–272]	0.826
> 15% from baseline	25 [3- 118]	49 [5–83]	0.995
SptO_2_			
Baseline; %	85 (5)	82 (8)	0.180
AUT 10% below baseline; %min	69 [1–363]	68 [5–498]	0.854

*SrtO*_*2*_ renal region tissue oxygenation, *SctO*_*2*_ cerebral tissue oxygenation, *SptO*_*2*_ peripheral tissue oxygenation, *AUT* area under the threshold. Significance is indicated with * for p < 0.05

### ROC analyses

The AUT SptO_2_ > 10% decrease from baseline showed the highest area under the ROC (AUROC) of 0.767 (95%CI = 0.691 to 0.914; p = 0.010) for predicting postoperative renal impairment (Table 4). The optimal cut-off SptO_2_ was 65%min with a sensitivity of 91% and a specificity of 63%. None of the thresholds set for renal region or cerebral tissue deoxygenation was able to predict postoperative renal impairment, nor was renal region specific tissue deoxygenation. Of note, the preoperative creatinine levels were also not predictive of postoperative renal impairment (AUROC of 0.508 (95%CI  0.334 to 0.864; p = 0.941).

**Table 4:** Results of ROC analyses

	AUROC	95% CI	P value
SrtO_2_			
Baseline, %	0.439	0.225 to 0.653	0.556
AUT 90%	0.597	0.366 to 0.828	0.346
AUT 80%	0.588	0.363 to 0.813	0.393
AUT 70%	0.612	0.407 to 0.817	0.276
AUT 60%	0.626	0.415 to 0.837	0.222
AUT > 5% from baseline	0.539	0.341 to 0.737	0.702
AUT > 10% from baseline	0.527	0.323 to 0.731	0.791
AUT > 15% from baseline	0.533	0.327 to 0.739	0.746
Renal region specific tissue deoxygenation			
AUT of renal specific tissue deoxygenation	0.433	0.232 to 0.634	0.517
SctO_2_			
Baseline, %	0.453	0.257 to 0.649	0.648
AUT 90%	0.676	0.472 to 0.880	0.088
AUT 80%	0.648	0.448 to 0.849	0.149
AUT 70%	0.591	0.381 to 0.801	0.377
AUT 60%	0.605	0.406 to 0.804	0.310
AUT > 5% from baseline	0.561	0.354 to 0.767	0.556
AUT > 10% from baseline	0.591	0.391 to 0.791	0.377
AUT > 15% from baseline	0.592	0.399 to 0.786	0.369
SptO_2_			
Baseline, %	0.442	0.257 to 0.628	0.576
AUT > 10% from baseline	0.767	0.619 to 0.914	0.010*
Preoperative serum creatinine, μmol l^−1^	0.508	0.334 to 0.864	0.941

*SrtO*_*2*_ renal region tissue oxygenation, *SctO*_*2*_ cerebral tissue oxygenation, *SptO*_*2*_ peripheral tissue oxygenation, *AUT* area under the threshold. Significance is indicated with * for p < 0.05

## Discussion

We did not observe an association between tissue oxygenation measured in the renal region or cerebral oxygenation and postoperative renal impairment in this small retrospective study. Surprisingly, a decrease in peripheral tissue oxygenation could predict postoperative renal impairment reasonably.

AKI frequently occurs after cardiac surgery [1, 2]. In contrast to established AKI clasifications, postoperative renal impairment is more subtle, yet even these subtle changes in serum creatinine levels are associated with increased postoperative morbidity and mortality [4, 5]. Therefore, it is important to assess the ability of different tools to predict impaired postoperative renal function and define patients at risk. Impaired postoperative renal function might be caused by ischemia or hypoxia during the intra-operative phase [17, 18]. Thus, it could be hypothesized that renal region tissue oxygenation monitoring by NIRS would be a useful predictor of postoperative renal impairment [10]. Several studies applying NIRS to the renal region in paediatric patients during cardiac surgery showed that decreased SrtO_2_ correlates with adverse outcomes and risk of renal impairment after cardiac surgery [11–15]. A possible explanation for the difference in predictive abilities between the paediatric and adult patients might be the positioning of the kidneys, which are closer to the skin in children. Another possible explanation is the different underlying morbidity in children. In a similar study of adult patients who underwent elective valve surgery, an absolute decrease in SrtO_2_ below 55% correlated significantly with the risk of postoperative renal impairment [19]. However, an absolute decrease below 55% hardly ever occurred in our population, which might indicate that the type of cardiac surgery could be an important factor in the development of postoperative renal impairment. An important difference between both studies is that we used renal depth > 5 cm as an exclusion criterion in our study instead of > 4 cm used in this study [19], which could also explain the different results.

In the past it has been suggested that cerebral oxygenation not only reflects oxygen status of the brain, but might also be an indication of oxygenation status in the entire body [20]. Cerebral oxygen saturation levels < 50% in cardiac surgery patients were found to be correlated with all-cause morbidity and mortality, in addition to cerebrovascular risk [21]. Therefore, we also assessed the predictive abilities of cerebral oxygenation. In our limited population we found no association between postoperative renal impairment and deoxygenation between absolute or relative thresholds of cerebral tissue deoxygenation. However, the 90% AUT absolute decrease in SctO_2_ almost reached statistical significance (p = 0.051), when compared between patients with and without postoperative renal impairment (Table 2). Hence, it may be speculated that if a larger sample size was observed, this association would prove to be statistically significant. Current evidence for predicting AKI with cerebral oxygenation is inconsistent. In one study assessing cerebral oxygenation in 150 adult patients undergoing cardiac surgery under CPB, cerebral oxygenation could not predict postoperative AKI [22], confirming our results. On the other hand, in a study including 59 paediatric cardiac surgery procedures, cerebral oxygenation was found to be a good predictor of renal replacement therapy with an AUROC of 0.866 (95%CI 0.770 to 0.961) [23]. One must note that this population was remarkably different regarding anatomy and type of surgery than the population we studied. However, in another study involving 45 adult patients undergoing cardiac surgery under CPB, an association was found between lower values of cerebral oxygenation and higher incidence of AKI (odds ratio, 0.667; 95% confidence interval, 0.485–0.917; p = 0.013) [24]. Of note, both studies including adult patients were conducted solely in on-pump cardiac surgery and not in off-pump procedures.

In contrast to renal region and cerebral tissue oxygenation, the AUT of a decrease from baseline > 10% of peripheral tissue oxygenation at the thenar muscle did appear to be a reasonable predictor of postoperative renal impairment. This finding could be well explained by assuming that we did not actually measure renal tissue oxygenation, rather than surrounding tissues as well. It is possible that peripheral tissue oxygenation provides a better indication of ‘general’ tissue oxygenation status. One of the aforementioned studies compared regional oxygen saturation in the thigh, forehead and abdomen also found that tissue oxygenation of the thigh was an independent risk factor for developing AKI [22], indeed suggesting that somatic oxygen saturation might provide a better indication of ‘general’ tissue oxygenation status. However, further research is needed to address this finding.

Since we studied a group of patients randomized to CABG performed with or without CPB, we were able to compare oxygenation data not only based on the development of renal impairment, but also between type of surgery. In our study we did not find any differences in renal region, cerebral and peripheral oxygenation between on-pump and off-pump CABG. However, the burden of renal region specific tissue deoxygenation was significantly higher in patients undergoing on-pump CABG. As mentioned before, CPB can cause disturbances in renal blood flow autoregulation and thus perfusion [8]. Additionally, the non-pulsatile flow usually used during CPB might hamper vascular responses to altered renal blood flow and consequently cause damage to renal tubules and glomeruli [25]. The literature contains conflicting data on the influence of surgical techniques on renal outcomes. Although a meta-analysis showed that off-pump CABG (compared with on-pump CABG) was not associated with improved renal outcomes [26], a large RCT including 4,752 patients undergoing isolated CABG in 19 countries showed that the use of off-pump CABG reduced the risk of postoperative AKI compared to on-pump [27].

Lastly, postoperative renal impairment might be associated with a longer duration of surgery and a larger number of grafts created (2 grafts (n = 7), 3 grafts (n = 20), 4 grafts (n = 12), 5 grafts (n = 2)). The duration of surgery and the number of grafts were significantly correlated in our study (r = 0.330). No differences were found between the number of grafts and renal region and peripheral oxygenation, except for the difference in AUT SptO_2_ > 10% from baseline (p = 0.02), which increased with the number of grafts created. The duration of surgery as possible risk factor for renal impairment was already suggested previously [28].

In our study we defined baseline values of oxygenation as the mean of those recorded during the 5-min interval prior to incision. Post-induction baseline values might be a better representation of physiologic conditions during surgery than awake baseline values and might therefore be a more realistic basis on which to determine treatment goals [29]. It could be argued that the most substantial part of the damage to the kidneys will occur intraoperatively rather than during induction, and therefore deviations in oxygenation below a stable post-induction level height of oxygenation may be more relevant. In a study, already mentioned above, SrtO_2_ data was recorded from pre-induction until the end of procedure, and this data showed only minor differences in oxygenation between the pre- and post-induction periods [19]. Additionally, the SrtO_2_ data from this study showed that the most significant decreases in SrtO_2_ occurred during surgery and further that the extent of the SrtO_2_ decrease was related to the duration of surgery.

One of the limitations of this study is the small sample size, which was caused by the design of the original study (pilot study) and the necessity to exclude 18 patients due to missing data. Moreover, only a few patients developed postoperative renal impairment. Another limitation is that the depth of the kidney was not recorded systematically. Therefore, it may be possible that in some of the patients, oxygenation of more superficial tissue was measured instead of the renal tissue. This may partly be responsible for the lack of predictive abilities from renal region oxygenation. In the original study, patients undergoing low-risk cardiac surgery were included and therefore the results cannot be extrapolated to the entire population undergoing cardiac surgery. For future research it would be interesting to have larger sample sizes and to include patients undergoing different types of cardiac surgery and patients having comorbidities in order to verify the generalizability of these results, for which the current observations might serve as reference. It would also be interesting to include awake baseline values and expand the monitoring period to the postoperative phase.

In conclusion, we did not observe an association between tissue oxygenation measured in the renal region and cerebral oxygenation and postoperative renal impairment in this small retrospective study. Instead, peripheral tissue deoxygenation was able to predict postoperative renal impairment, suggesting that monitoring peripheral tissue oxygenation provides a better indication of ‘general’ tissue oxygenation status.

## Data Availability

Data and materials are available upon request.
