# Correction to: Comparison of renal region, cerebral and peripheral oxygenation for predicting postoperative renal impairment after CABG

**DOI:** 10.1007/s10877-021-00791-0

**Published:** 2021-12-16

**Authors:** Ilonka N. de Keijzer, Marieke Poterman, Anthony R. Absalom, Jaap Jan Vos, Massimo A. Mariani, Thomas W. L. Scheeren

**Affiliations:** 1 Department of Anaesthesiology, University Medical Centre Groningen, Hanzeplein 1, Groningen, 9713 GZ The Netherlands; 2 Department of Cardiothoracic Surgery, University Medical Centre Groningen, Groningen, The Netherlands

## Correction to: Journal of Clinical Monitoring and Computing 10.1007/s10877-021-00701-4

In the original publication of the article, the 95% CI of peripheral tissue oxygenation decrease > 10% from baseline was published incorrectly under Abstract section. The correct CI should read as 95% CI 0.619 to 0.914; p = 0.010.

